# A novel study of screening and confirmation of modafinil, adrafinil and their metabolite modafinilic acid under EI-GC-MS and ESI-LC-MS-MS ionization

**DOI:** 10.4103/0253-7613.59928

**Published:** 2009-12

**Authors:** S. Dubey, S. Ahi, I. M. Reddy, T. Kaur, A. Beotra, S. Jain

**Affiliations:** National Dope Testing Laboratory, Ministry of Youth Affairs and Sports, J. N. Stadium, New Delhi - 100 03, India

**Keywords:** Adrafinil, EI-GC-MSD, ESI-LC-MS/MS, modafinil, modafinilic acid

## Abstract

**Objective:**

Adrafinil and modafinil have received wide publicity and have become controversial in the sporting world when several athletes were discovered allegedly using these drugs as doping agents. By acknowledging the facts, the World Anti-Doping Agency (WADA) banned these drugs in sports since 2004. The present study explores the possibility of differentiating adrafinil and modafinil and their major metabolites under electron impact ionization in gas chromatograph–mass spectrometer (GC-MSD) and electrospray ionization in liquid chromatograph–mass spectrometer (LC-MS/MS) by studying the fragmentation pattern of these drugs.

**Materials and Methods:**

Adrafinil, modafinil and their major metabolite, modafinilic acid were analyzed on EI-GC-MSD and ESI-LC-MS/MS using various individual parameters on both the instruments. The analytical technique and equipment used in the analysis were an Agilent 6890N GC with 5973 mass selective detector for the GC-MSD analysis and an Agilent 1100 HPLC with API-3200 Triple quadrupole mass spectrometer for the LC-MS/MS analysis. Validation of both methods was performed using six replicates at different concentrations.

**Result and Discussion:**

The results show that adrafinil, modafinil and their major metabolite modafinilic acid could be detected as a single artifact without differentiation under EI-GC-MSD analysis. However, all drugs could be detected and differentiated under ESI-LCMS/MS analysis without any artifaction. The GC-MSD analysis gives a single artifact for both the drugs without differentiation and thus can be used as a marker for screening purposes. Further, the Multiple Reaction Monitoring (MRM) method developed under LC-MS/MS is fit for the purpose for confirmation of suspicious samples in routine sports testing and in forensic and clinical analysis.

## Introduction

Adrafinil and modafinil are clinically used in the treatment of narcolepsy, obstructive sleep apnea and idiopathic hypersomnia. Modafinil is a central nervous system stimulant, which possess wake-promoting actions like symathommimetic agents, including amphetamine and methylphenidate. Adrafinil is a prodrug of modafinil and is readily converted into modafinil and its metabolite modafinilic acid after intake. These drugs have received wide publicity and become controversial in the sporting world when several athletes were discovered allegedly using them as doping agents. By acknowledging the facts, the World Anti-Doping Agency (WADA) banned these drugs in sports from 2004.[1] The minimum required performance limit for both modafinil and adrafinil is 500 ng/ml.[1] The testing for modafinil and adrafinil in clinical, forensic and dope testing was being conducted by gas chromatograph–mass spectrometer (GC-MSD)[2] and high-performance liquid chromatography (HPLC).[3,4] However, during the analysis of all the three drugs, viz. adrafinil, modafinil and modafinilic acid, it was not possible to differentiate them on GC-MSD since they give a single peak. Moreover, HPLC analysis possesses the shortcoming of quantitation analysis for all the three compounds. Hence, liquid chromatograph–tandem mass spectrometer (LC-MS/MS) becomes the method of choice for detection of either of the drugs or metabolites. The present study explores the possibility of differentiating adrafinil, modafinil and their major metabolites by LC-MS/MS. A method was developed for screening on GC-MS and confirmation on LC-MS/MS for adrafinil, modafinil and modafinilic acid and validated as per the international guidelines.[5,6]

## Materials and Methods

### Reference standards

The reference standards of modafinil and adrafinil were purchased from Sigma (St-Louis, MO, USA). The organic solvents and reagents were of HPLC grade. The Amberlite XAD-2 was purchased from Sigma-Aldrich (St. Louis, MO, USA) and the derivatizing reagent, iodomethane was purchased from Acros Organics (New Jersey, USA). Acetonitrile and ethyl acetate were obtained from Qualigens (Mumbai, India), methanol from J.T Baker (Phillipsburg, NJ USA), tertiary butyl methyl ether (TBME) from Acros Organics and formic acid from Merck (Mumbai, India). deionised water was prepared on a Milli Q laboratory plant (Millipore, Bedford, MA, USA).

### Sample extraction procedure for GC-MSD analysis

The sample extraction procedure for GC-MS analysis involves solid phase extraction.

Two milliliters of urine was applied onto the pre-prepared XAD_2_ column. Mefruside (2 *μ*g/ml) was added as the internal standard. Washing was performed with 2 ml of water to eliminate most of the water-soluble urinary constituents that had not been absorbed on the solid support. The drugs were then eluted with 2 ml of methanol. The entire effluent was evaporated under nitrogen stream at 60° C and the residue was dissolved in 200 *μ*l acetone and 50 mg K_2_CO_3_ was added to make the reaction mixture alkaline. Then, derivatization was performed using iodomethane for 3 h at 60° C. The acetone layer was then cooled and dried under nitrogen evaporator at 60°C and the sample was reconstituted in 50 *μ*l of ethyl acetate and injected on GC-MSD.

### Sample extraction procedure for LC-MS/MS analysis

The sample extraction procedure used for LC-MS/MS involves liquid-liquid extraction. Two/four milliliters of the urine sample based on specific gravity was taken and 500 ng/ml of methyl testosterone was added as the internal standard. One milliliter of phosphate buffer was added to adjust the pH to 7.0. Hydrolysis was performed by addition of ß-glucuronidase enzyme (*E. coli*) to the sample. The sample was incubated at 60°C for 1 h and 250 *μ*l of K_2_CO_3_ (pH 9-10) was added. Liquid-liquid extraction was performed by addition of TBME and the organic layer was separated in another test tube. To the remaining aqueous layer, 150 *μ*l of 6N HCl (pH 2-3) and 4 ml of ethyl acetate were added. A second liquid-liquid extraction was performed and the organic layer was separated into the initial test tube. The collective organic layers of both the extraction steps were evaporated to dryness. Finally, the dry extract was reconstituted in 100 *μ*l of the mobile phase (50:50, V/V) injected on LC-MS/MS.

### Instrumentation and conditions

## GC-MSD

Gas chromatography mass spectrometric analysis was carried out on an Agilent 6890N Network GC system with 5973 Network mass selective detector, equipped with 7683 series automated liquid sampler (Agilent Technologies Inc., Wilmington, DE, USA). The entire system is controlled by the Chemstation® Software (Agilent Technologies Inc.). The instrumental conditions are shown in Table 1.

**Table 1:** Analytical parameters of GC-MSD

Injection mode	Automatic split
Split ratio	11:1
Injection volume	2 μl
Injection port temperature	280°C
Carrier flow	135 kpa helium (constant pressure)
Oven program	150°C for 1 min, 19.5°C/min, 300°C final hold for 2 min
Column	Ultra 2, fused silica, 0.2 mm × 12.5 m × 0.11 μm

## LC-MS/MS

An Aglient 1100 series LC system coupled with API 3200 Triple quadrupole instrument equipped with a pneumatically assisted electrospray ion source (Applied-Biosystem-Sciex Concord, Canada) was used. The entire system was controlled using the Analyst 1.4.1® software. The main working parameters of the mass spectrometer are summarized in Table 2.

**Table 2:** Analytical parameters of LC-MS/MS

Column	Inertsil C-18 column (3.0 μm × 50 mm × 4.6 mm)
Flow	700 μl/min
Mobile phase	A: 1% formic acid; B: acetonitrile
Gradient	0-5 min B 15%, 5-6 min B 60%, 6-7 min B 100%, 7-11 min B 15%
Polarity	Positive
Source	550°C
Curtain gas	15 psi
CAD	3 psi

## Method development on GC-MSD and LC-MS/MS

The method development was initiated by direct derivatization and injection of the reference standards of both adrafinil and modafinil on GC-MSD. For modafinilic acid, the excretion study sample was processed and injected in scan mode and the method was developed. The scan range was 40-550 with a scan rate of 2.8 scan/second with an injection volume of 2 *μ*l.

For the LC-MS/MS analysis, direct infusion of individual reference standards dissolved in ethanol was performed using different collision energies. Ionization in the turbospray source was performed in positive mode scanning masses from m/z 100 to 500 with a 0.2 *μ* step size. Nitrogen was used as nebulising and curtain gas. For modafinilic acid, the excretion study sample was processed and injected through LC in scan mode and the method was optimized.

## Method validation

The analytical method was validated as per the requirement of WADA ISL (version 6.0) keeping in view linearity, accuracy, precision, specificity, recovery, limit of detection (LOD) and limit of quantitation (LOQ).

Working solutions for calibration and quality control samples were prepared from 1 mg/ml of stock solution by dilution using ethanol for GC-MS and LC-MS/MS, separately. Quality control samples (spiked) were prepared alike in six replicates at four concentrations, i.e. 100 ng/ml, 250 ng/ml, 500 ng/ml and 1000 ng/ml for LC-MS/MS analysis and six replicates at three concentrations, i.e. 250 ng/ml, 500 ng/ml and 1000 ng/ml for GC-MS analysis. The concentrations of the direct calibration standard were 100 ng/ml, 250 ng/ml, 500 ng/ml and 1000 ng/ml.

## Excretion study of modafinil

Two healthy male volunteers aged 25 ± 3 years were given one single dose of modafinil (100 mg; Sun Pharmaceuticals, Vapi, Gujarat, India) as per the approval of the Ethics Committee. Urine samples were collected for 72 h and were stored at −20°C.

## Results

**Figure 1:**
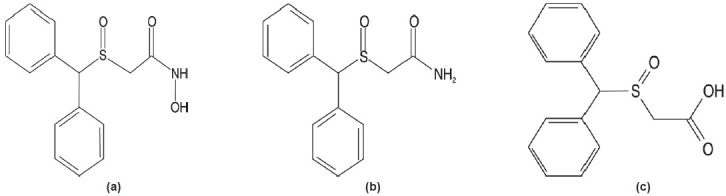
Chemical structure of (a) adrafinil, (b) modafinil and (c) modafinilic acid

**Figure 2:**
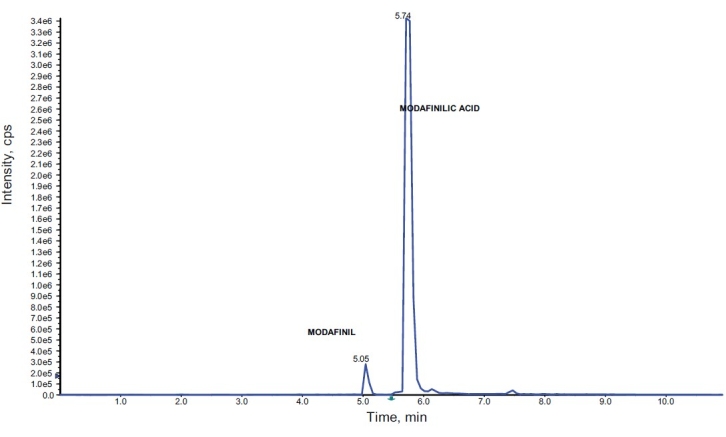
Total ion chromatogram of an excretion study sample of adrafinil analyzed on ESI-LC-MS/MS in MRM mode showing presence of adrafinil, modafinil and modafinilic acid

Behavior of modafinil, adrafinil and modafinilic acid under EI-GC-MS and ESI-LCMS/MS:

2-[(diphenylmethyl) sulfinyl] acetamide (modafinil) and 2-[(diphenylmethyl) sulfinyl] hydroxamide (adrafinil) and their carboxylic metabolite modafilinic acid are severely degraded during EI-GC-MS analysis. Due to the sterically rigid and strong electronegative structure, the only possible site of ionization is the diphenylmethyl sulfinyl linkage Hence, as expected from the structures of adrafinil, modafinil and their metabolite modafinilic acid as shown in Figure 1, the main fragment comes from diphenylmethyl sulfinyl linkage giving m/z 167 as the most prominent ion. Further, demethylation occurs from this fragment giving m/z 152 as second main fragment. The high temperature programming used in gas chromatography leads to the formation of a single artifact of modafinil, adrafinil and modafinilic acid under which they elute as a single peak at the same retention time. Whereas, in liquid chromatography condition, due to lack of high temperature programming these compounds are not degraded and therefore show good separation and resolution with different retention times [Figure 2]. The comparative result for the analysis of modafinil, adrafinil and modafinilic acid under EI-GC-MS and ESI-LC-MS/MS are tabulated in Table 3.

**Table 3:** Retention time, mass fragments (m/z) and MRM transitions of adrafinil, modafinil and modafinilic acid on GC-MSD and LC-MS/MS

*Compound*	*Retention time (min)*		*MRM transitions*	*Fragmentation ions*
			
	*(GC-MSD)*	*(LC-MS/MS)*	*(LC-MS/MS)*	*(GC-MSD)*
Adrafinil	7.2	4.9	288–104	167, 165, 152
Modafinil		5.1	274-167	167, 165, 152
Modafinilic acid		5.8	273-167	167, 165, 152

### Limit of detection

To measure the limit of detection for adrafinil and modafinil, six aliquots of negative urine samples were spiked with 100 ng/ml and 250 ng/ml of certified reference standard for GC-MS analysis and 25 ng/ml, 50 ng/ml and 100 ng/ml of certified reference standard for LC-MS/MS analysis. The LOD for modafinil and adrafinil was found to be 250 ng/ml on GC-MS and 100 ng/ml on LC-MS/MS.

### Calibration curve

**Figure 3:**
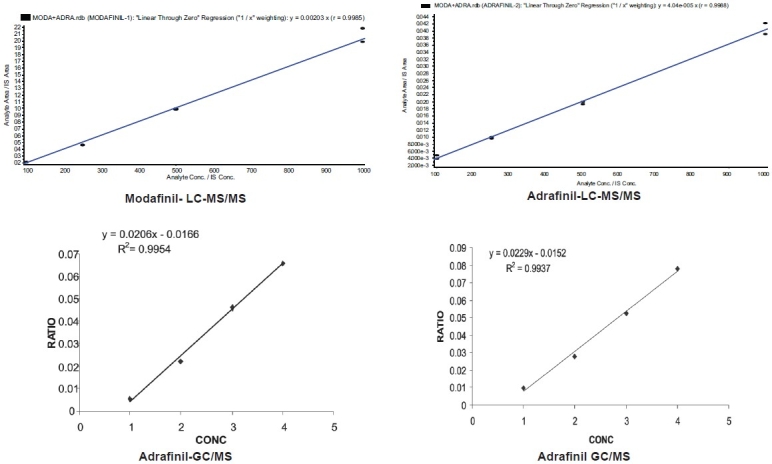
Calibration curves of modafinil and adrafinil on GC-MS and LC-MS/MS

A calibration curve was constructed with different concentrations ranging between 100 ng/ml and 1000 ng/ml on GC-MSD and LC-MS/MS for both modafinil and adrafinil. Linearity was assessed by a weighted (1/x) least squares regression analysis. The calibration curve had a correlation coefficient (r^2^) of ≥0.99 of both the drugs on the GC-MSD and LC-MS/MS instruments. The acceptance criterion for each calculated standard concentration was 15% deviation from the nominal value [Figure 3].

### Precision and accuracy

The batch accuracy and precision were determined by analyzing six sets of quality control samples. The accuracy of detection of modafinil and adrafinil were in the range of 86-88% and 94-104% on GC-MS and LC-MS/MS, respectively. The precision of the method was expressed as CV%. Tables 4 and 5 summarize the calculated concentrations, recovery percentage, accuracy and precision of the method for spiked samples of adrafinil and modafinil in human urine on GC-MS and LC-MS/MS.

**Table 4:** Recovery percentage, accuracy and precision of modafinil and adrafinil on GC-MS

*Compound*	*Expected concentration (ng/ml)*	*Calculated concentration (ng/ml) (mean ± SD)*	*Recovery percentage (mean ± SD)*	*CV%*	*Accuracy %*
Modafinil (n = 6)	250	219 ± 22.4	80.4 ± 8.5	10.2	87.6
	500	422 ± 32.4	69.2 ± 5.3	7.6	84.4
	1000	868 ± 100.4	97.7 ± 11.2	11.5	86.8
Adrafinil (n = 6)	250	219 ± 22.4	68.6 ± 6.5	10.2	87.6
	500	422 ± 32.4	71.2 ± 4.6	7.6	84.4
	1000	868 ± 100.4	81.6 ± 9.4	11.5	86.8

**Table 5:** Recovery percentage, accuracy and precision of modafinil and adrafinil on LC-MS/MS

*Compound*	*Expected concentration (ng/ml)*	*Calculated concentration (ng/ml) (mean ± SD)*	*Recovery percentage (mean ± SD)*	*CV%*	*Accuracy %*
Modafinil (n = 6)	100	101 ± 5.4	101.2 ± 5.4	5.3	101
	250	240 ± 16.8	102.3 ± 7.1	7.0	96.5
	500	519 ± 24.0	106.4 ± 4.9	4.6	103.8
	1000	1026 ± 39.9	99.6 ± 3.8	3.8	102.6
Adrafinil (n = 6)	100	88 ± 4.5	89.2 ± 4.5	5.1	88.6
	250	236 ± 10.8	100.7 ± 4.6	4.6	94.4
	500	483 ± 16.4	97.9 ± 3.3	3.3	96.6
	1000	1042±40.8	101.7±3.9	3.9	104.2

### Specificity and matrix effect

**Figure 4:**
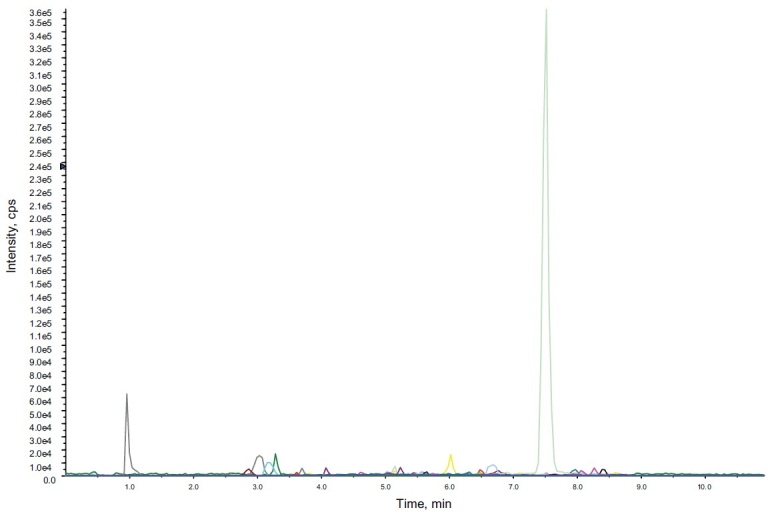
Total ion chromatogram of a negative urine sample analyzed on ESI-LC-MS/MS in MRM mode showing absence of adrafinil, modafinil and modafinilic acid

The specificity and matrix effect of the method was examined on both the instruments by analyzing the drug free urine processed through the sample procedure as applied for the quality control samples. No major interference peaks were observed on either of the instruments [Figure 4].

### Selectivity

The selectivity of the method was examined by analyzing the drug free urines fortified with the drugs having properties similar to adrafinil and modafinil at various concentrations. No compound has shown any co-elution or interference with mass spectra of the analyte of interest on both the instruments.

### Recovery

Recoveries of adrafinil, modafinil and internal standard were evaluated by comparing the mean peak areas of the processed samples (spiked) with the mean peak areas of the unprocessed direct reference standard solutions of the same concentration. Recoveries of all compounds were found to be within the acceptable range [Tables 4 and 5]. Internal standards are good and acceptable for both the methods.

### Excretion study sample

**Figure 5:**
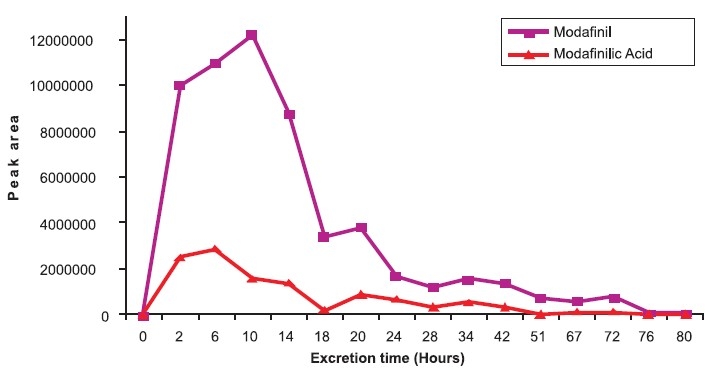
Excretion profiles of modafinil and modafinilic acid in a human male volunteer

Modafinil and its main metabolite, modafinilic acid were identified in all the excretion study samples. But, the metabolite could not be quantitated due to unavailability of the pure standard. A graph was plotted for area of abundance against excretion time to explore the excretion profile of the parent and metabolite. Parent drug could be traced till eighty hours post drug administration, whereas the peak concentration was eliminated within 6 h [Figure 5].

## Discussion

The present method developed shows that adrafinil, modafinil and their major metabolite, modafinilic acid could be detected as a single artifact without differentiation under EI-GC-MSD analysis. However, all drugs could be detected and differentiated under ESI-LC-MS/MS analysis without any artifaction at different retention times. The GC-MSD analysis gives a single artifact and thus can be used as a marker for screening purposes. Further, ESI-LC-MS/MS allows the differentiation of adrafinil, modafinil and modafinilic acid; hence the method is appropriate for the purpose for confirmation of suspicious samples in routine sports testing and in forensic and clinical analysis. Modafinil and adrafinil are the stimulants with the potential of abuse in sports.[1] Modafinil is approved by the FDA for use in the management of excessive sleepiness associated with narcolepsy.[7] This method can be of utility for detecting and differentiating modafinil, adrafinil and modafinilic acid. It is free from interference by methylphenidate, cocaine, amphetamines and other drugs of abuse. The quantitation of excretion study samples shows that peak levels of modafinil could be found at 10 h both in GC-MS and LC-MS/MS analysis, which proves the authenticity of the method.

The pharmacological properties of modafinil and adrafinil are similar to amphetamine but without some of the side-effects associated with amphetamine like stimulants. Hence, both modafinil and adrafinil have very good potential to be abused, thereby necessitating the need to have screening and confirmation method. Further work is in progress to differentiate enantiomers of modafinil on LC-MS/MS.
